# Novel RNA-methylase HNRNPC promotes gastric cancer tumorigenesis by triggering the lactate-induced ferroptosis resistance

**DOI:** 10.3389/fimmu.2025.1612935

**Published:** 2025-09-04

**Authors:** Guoqiang Yang, Lei Shen, Mengqian Cui, Jian Yang

**Affiliations:** Department of Gastroenterology, Zibo Central Hospital, Zibo, China

**Keywords:** ferroptosis, HNRNPC, lactate, MCT1, gastric cancer

## Abstract

**Background:**

Emerging evidence gradually indicates that lactate and iron-induced cell death plays important role in gastric cancer (GC) progression. Here, this study focused on the effect of ferroptosis-related N^6^-methyladenosine (m^6^A) modification on GC progression.

**Methods:**

The ferroptosis-related characteristic and lactate were tested by the kits. The in vivo mice animal assay was performed by subcutaneous xenotransplantation. The glycolysis-related analysis was performed by extracellular acidification rate (ECAR) and oxygen consumption rate (OCR) analysis.

**Results:**

The elevated Heterogeneous Nuclear Ribonucleoprotein C (HNRNPC) expression positively fortified the aerobic glycolysis and lactate accumulation in GC. The exogenous lactate accelerated the proliferation, oxaliplatin resistance and aerobic glycolysis in GC that inhibited by HNRNPC silencing. Moreover, HNRNPC silencing up-regulated the iron concentration accumulation and ferroptosis, and the exogenous lactate and ferrostatin-1 (Fer-1, ferroptosis specific inhibitor) co-administration reduced the iron concentration. Mechanistically, MCT1 was identified as the downstream target of HNRNPC, and HNRNPC targeted MCT1 to fortify the lactate accumulation, thereby accelerating the ferroptosis resistance in GC.

**Conclusions:**

Overall, these findings revealed the novel role of ferroptosis-related HNRNPC on GC lactate accumulation and lactate-induced tumorigenesis in GC tumor microenvironment. The data revealed the importance of HNRNPC for lactate metabolism in GC tumor microenvironment, as well as the synergistic effect of HNRNPC on lactate-induced ferroptosis resistance.

## 1 Introduction

In recent years, advancements in the clinical treatment of gastric cancer (GC) have been notable (1). For GC. the primary treatment modalities include surgical resection, chemotherapy, radiotherapy, and targeted therapy. Surgical resection remains the preferred approach for early-stage GC, with reduced invasiveness and complications due to the advancements in laparoscopic and robotic surgery techniques (2). For intermediate and advanced stages, the combination of treatment options has significantly enhanced patient survival rates, particularly with the utilization of novel chemotherapy and targeted drugs for locally advanced and metastatic cases. Despite these improvements, the overall prognosis for GC remains suboptimal, particularly in advanced scenarios, with low five-year survival rates (3). Moving forward, the exploration of new biomarkers and molecular targets is essential to improve treatment outcomes and enhance patient quality of life.

N^6^-methyladenosine (m^6^A) is the most common mRNA post-transcriptional modification, mediating more than 60% of RNA methylation (4, 5). Abnormal m^6^A methylation levels are closely related to cell differentiation and immune function, and play an important role in the progression of various cancers. The frequency of m^6^A methylation changes in cancer mainly depends on the expression of methylation regulators, such as methyltransferases, demethylases and readers. In the GC tumor progression, more and more m^6^A methylation regulators have been identified, such as METTL14 (6), METTL3 (7), WTAP (8), FTO (9), KIAA1429 (10), hnRNPA2B1 (11).

Lactate is an important by-product in tumor metabolism and the role of lactate in tumorigenesis has attracted more and more attention (12). Studies have shown that lactate promotes tumor cell invasion and metastasis by reducing local pH, and promotes tumor cell survival and proliferation by activating signaling pathways such as HIF - 1α and NF-κB (13). In addition, lactate can inhibit anti-tumor immune responses by reprogramming immune cell function, thus providing a favorable environment for tumor cells growth. In lactate transport, MCT1 is a critical protein found in monocarboxylate transporters that plays a significant role in regulating the lactate shuttle (14).

The roles of HNRNPC have been wildly reported, including cervical cancer (15), glioma (16), aging adipose tissue (17). In the study, the present research explored the role of m^6^A reader HNRNPC on the GC aerobic glycolysis, lactate and iron homeostasis. Results unveiled that HNRNPC positively fortified the lactate accumulation and ferroptosis resistance. One of the most interesting findings of this study was that exogenous lactate and ferroptosis specific inhibitor Ferrostatin-1 (Fer-1) co-administration could both reduce the iron concentration. HNRNPC regulates the lactate metabolism and ferroptosis in GC tumor microenvironment. These findings revealed the critical role of HNRNPC on GC lactate accumulation and ferroptosis resistance in GC tumor microenvironment.

## 2 Materials and methods

### 2.1 Cells culture, treatment and transfection

One gastric epithelial cell line (GES - 1) and three GC cell lines (MKN74, HGC27, AGS, SNU216) were selected from Chinese Academy of Sciences (Shanghai). MKN74 and HGC27 cells were RPMI - 1640 (cat No. CAT#01-100-1ACS, BI) containing 10% FBS. AGS cell was cultured in DMEM/F12 (cat No. CAT#01-172-1ACS, BI) + 10% FBS (cat No. CAT#SA102.02, Cellmax). The culture environment was 37 °C and 5% CO2 concentration. For the exogenous lactate treatment, the L-lactate (Sigma, L1750) was added to each cell group (20 mmol/L). Besides, the carcinoma-associated fibroblasts (CAFs)-conditioned medium (CM) was prepared for exogenous lactate treatment.

For the cell transfection, the lentiviral vectors for stable knockdown and overexpression of HNRNPC were provided by GeneChem (Shanghai, China). GC cells were seeded in 24-well plates, and then for transfection with the concentrated virus (MOI = 100) when cells reached 60%-70%. Then, the infected cells were treated with puromycin (Sigma-Aldrich, 2 μg/mL).

### 2.2 qRT-PCR analysis

Total RNA was prepared from cells using TRIzol (Tiangen, cat No. DP430) according to its instructions of manufacturer. The quality of RNA samples was evaluated via spectrophotometric analysis and then subjected to HiScript III 1st Strand cDNA Synthesis Kit (Vazyme, Shanghai, China) to generate cDNA. ChamQ SYBR qPCR Master Mix (Vazyme, Q311 - 02/03) was used for real-time PCR to determine the mRNA level relative to beta-actin by the 2^−ΔΔCt^ method. The sequences of primers used in this study were shown in **Supplementary Table S1**.

### 2.3 Single-cell RNA sequencing

Single-cell suspensions were prepared from fresh tissues using enzymatic digestion and mechanical dissociation. Cell viability (>85%) and concentration were confirmed via trypan blue staining and automated counting. Approximately 10,000 cells per sample were loaded onto a 10x Genomics Chromium Controller to generate single-cell Gel Bead-In-EMulsions (GEMs). Libraries were constructed using the Chromium Single Cell 3′ Reagent Kit v3.1, followed by paired-end sequencing (150 bp) on an Illumina NovaSeq 6000 platform at a depth of ~50,000 reads per cell. Raw sequencing data were processed via Cell Ranger (v7.1.0) for alignment, filtering, and UMI counting. Downstream analyses, including normalization, dimensionality reduction (PCA, UMAP), and cluster identification, were performed using Seurat (v5.0.1) and Scanpy (v1.9.3). Low-quality cells (mitochondrial genes >20% or UMIs <500) were excluded. Cell types were annotated via marker gene expression from public databases.

### 2.4 EdU and CCK - 8 assay

The proliferation of GC cells was tested by EdU and CCK - 8 assay. In brief, the transfected GC cells were utilized. EdU incorporation assay was carried out with an EdU kit (Roche, Mannheim, Germany). The oxaliplatin resistance was tested using CCK - 8 for the IC_50_ value (50% maximal inhibitory concentration).

### 2.5 Lactate analysis

For lactate production analysis, GC cells were cultured with a completed medium (24 h) and the culture medium was changed by fresh medium. After incubation (6 h) of cultured medium, the lactate content was detected by stable isotope tracing analysis (18) or L-Lactate Assay Kit (Colorimetric, ab65331, Abcam).

### 2.6 The extracellular acidification rate and oxygen consumption rate analysis

ECAR was determined using Seahorse XF96 Analyzer Glycolysis kit (Agilent Technologies, Cat No. 103344) and the OCR was detected using Seahorse XF Cell Mito Stress Test kit (Cat No. 103015). GC cells (10^4^) were inoculated into XF96 culture plates. Then, ECAR was determined after saturating concentration of glucose, oligomycin and 2-deoxyglucose (2-DG) addition at the indicated time points. OCR was determined by adding oligomycin, Trifluoromethoxy carbonylcyanide phenylhydrazone (FCCP) and Rotenone/antimycin A treatment. ECAR (mpH/min) and OCR (pmol/min) were automatically calculated via Seahorse XFp software.

### 2.7 Iron concentration, GSH and ROS analysis

The iron concentration was detected using the Cell Total Iron Colorimetric Assay Kit (Cat. E-BC-K880-M, Elabscience) according to the manufacturer’s instructions. The GSH was tested by commercialized GSH/GSSG assay kit (Beyotime, Cat. S0053). The lipid ROS level was tested by BODIPY™ 581/591 C11 reagent (Cat. D3861, Invitrogen, California, USA).

### 2.8 Transmission electron microscopy

GC cells were embedded and stained for the examining mitochondrial morphology. The sample images were observed under a transmission electron microscope (HT7700, HITACHI).

### 2.9 RIP assay

To confirm molecular interactions, RIP analysis was performed using a RNA Binding Protein Immunoprecipitation Kit (Magna, Shanghai, China). A total of approximately 10^7^ GC cells were collected and lysed. After removal of DNA, lysates were incubated with RIP buffer containing anti-HNRNPC antibody (Abcam, 1:1000, Cat No. ab314004) and normal control IgG (Bioss, Cat No. bs-0295PC) were incubated for 16 hours at 4°C. The RNA-protein complexes were then incubated with protein A/G balanced magnetic beads. After elution of RNA, the immunoprecipitated RNA was extracted for the A/G magnetic beads, and qRT-PCR was performed.

### 2.10 RNA decay analysis

Transfected GC cells were treated with 8 μg/ml actinomycin D (Act D) for 0, 3, 6, and 9 hours, respectively, and RNA was extracted for reverse transcription and qRT-PCR. Relative quantitation was calculated using the 2^-ΔΔCt^ method and normalized to β-actin. Calculation of the half-life of MCT1 mRNA was conducted.

### 2.11 Animal assay

A subcutaneous transplanted tumor model was established by subcutaneously injecting 100 μL of MKN74 cells suspension into the flank of BABL/c nude mice that housed in specific pathogen-free (SPF) animal facility. Tumor size was measured once three days, and the calculation formula was: volume = length×width^2^/2. Three weeks after injection, tumors were harvested, weighed, and stored for further study. All animal experiments were conducted in strict compliance with the National Institutes of Health Ethical Principles and Guidelines for the Care and Use of Animals. This study had been approved by the Committee on Animal Research of Zibo Central Hospital.

### 2.12 Statistical analysis

Statistical analysis was performed using GraphPad Prism 8.0 software and Student’s t test or ANOVA (multiple comparisons between multiple groups). Unless otherwise stated, data are expressed as mean ± standard deviation. Kaplan-Meier method and log-rank test were used for overall survival analysis. The p-value is 0.05, and the difference is statistically significant.

## 3 Results

### 3.1 Elevated HNRNPC expression in GC

**Figure 1:**
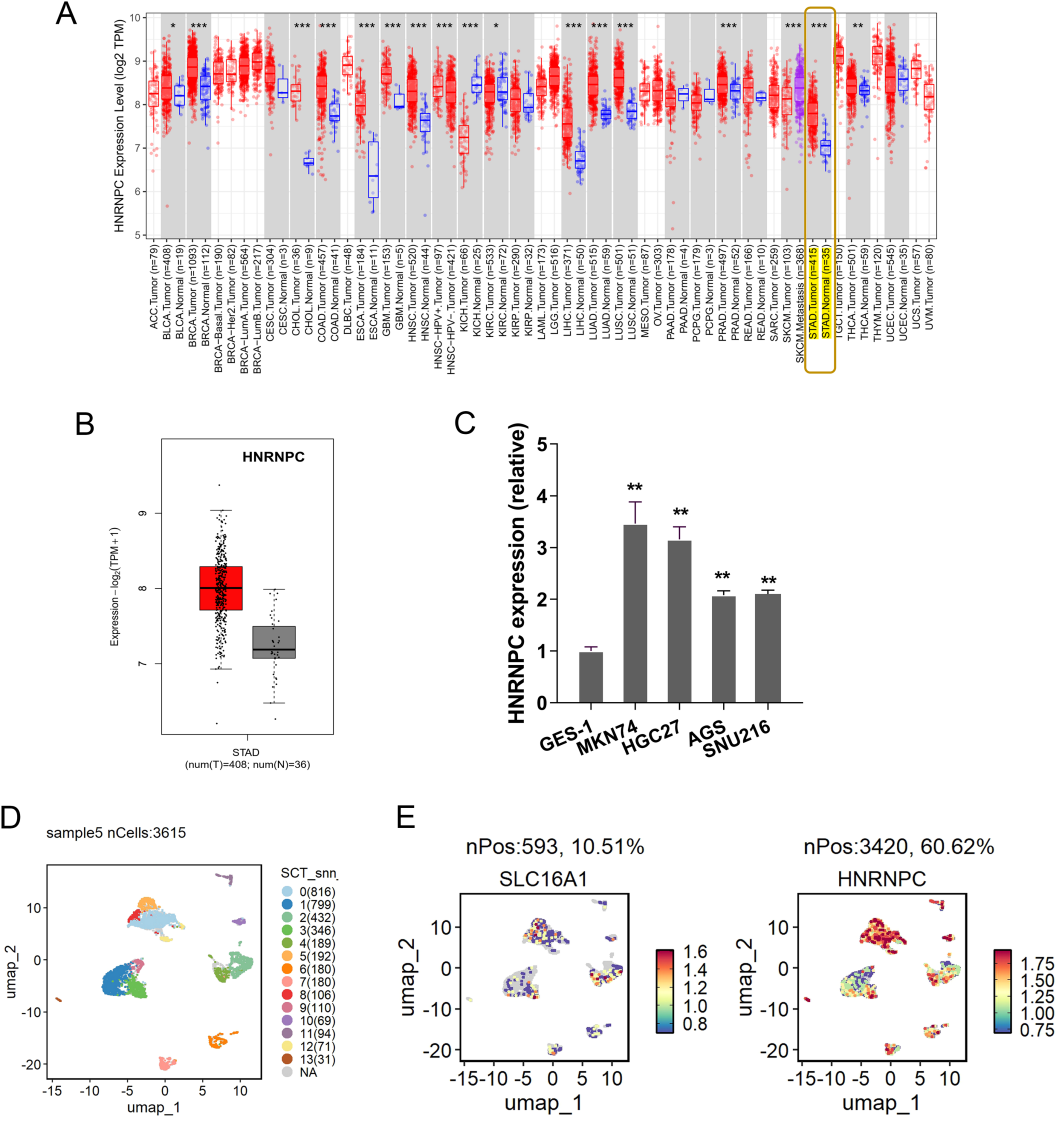
Elevated HNRNPC expression in GC. **(A)** The HNRNPC expression was tested in multitudinous human cancers, especially the GC (http://timer.cistrome.org/). **(B)** In the public dataset, the HNRNPC level significantly over-regulated as comparing to normal control group (http://gepia.cancer-pku.cn/index.html). **(C)** The HNRNPC level in GC cells (MKN74, HGC27, AGS). **(D)** The scRNA-seq in the GC samples (GSE183904). **(E)** SLC16A1 (MCT1) and HNRNPC high-regulation in the GC samples. **p<0.01. *p<0.05. ***p<0.001.

To investigate whether HNRNPC affects the GC progression, the HNRNPC expression was tested in multitudinous human cancers, and results illustrated that HNRNPC level significantly elevated, especially the GC (**Figure 1A**). The HNRNPC was a remarkable high-expression one as comparing to normal control group (**Figure 1B**). In GC cells, the HNRNPC level was found to be highly expressed (**Figure 1C**). Besides, the HNRNPC was also tested in the scRNA-Seq (**Figure 1D**). Results indicated that HNRNPC and SLC16A1 (MCT1) were both up-regulated in the GC samples (**Figure 1E**). Conclusively, the results obtained from this study affirmed the elevated HNRNPC expression in GC.

### 3.2 HNRNPC promoted the aerobic glycolysis and lactate accumulation in GC

**Figure 2:**
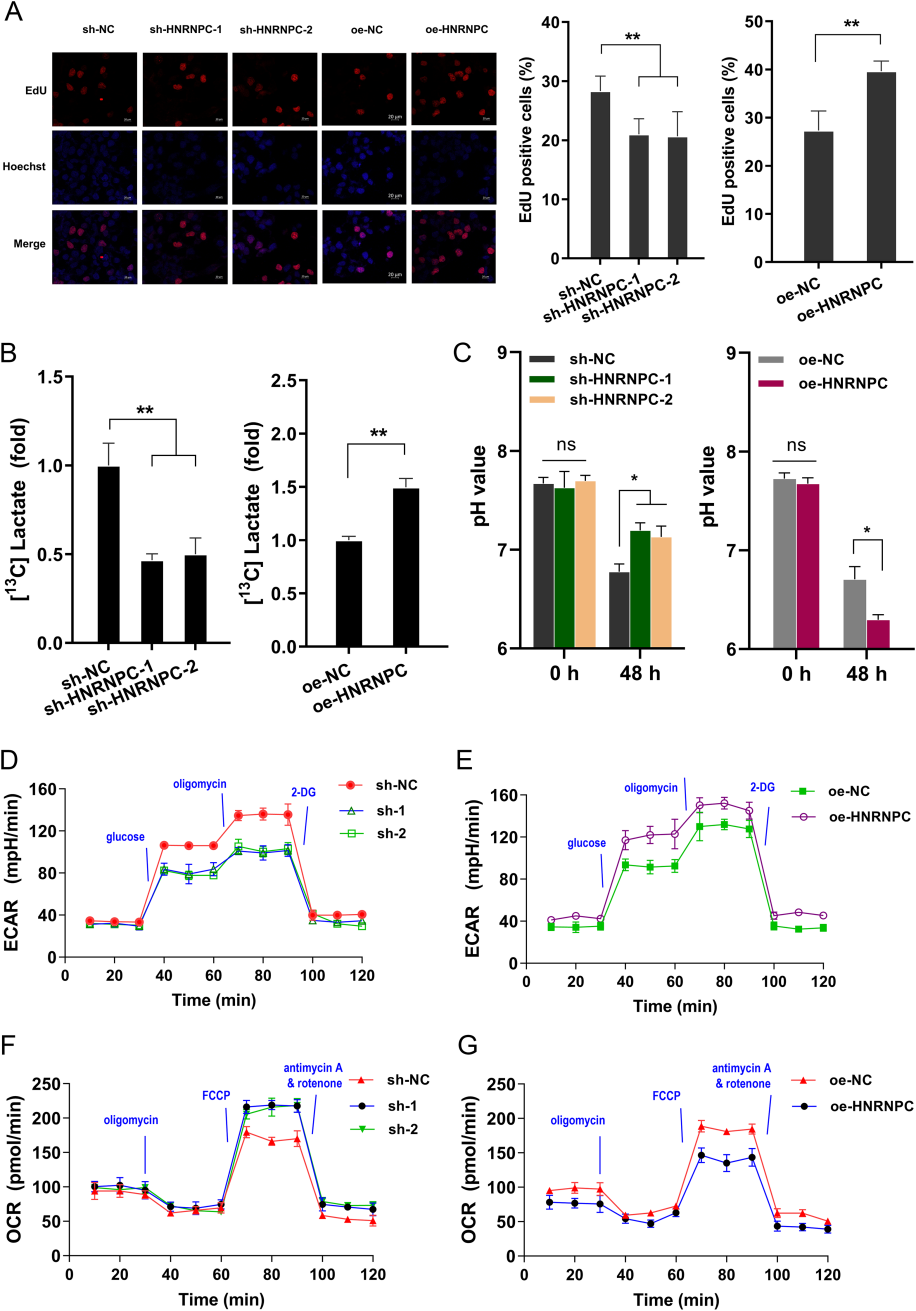
HNRNPC positively fortified the aerobic glycolysis and lactate accumulation in GC. **(A)** The proliferation assay by EdU assays indicated the proliferation of GC (MKN74) cells with stably HNRNPC silencing (sh-HNRNPC-1, sh-HNRNPC-2) and HNRNPC over-expression. **(B)** Lactate production was measured in MKN74 cells with stably HNRNPC silencing (sh-HNRNPC-1, sh-HNRNPC-2) and HNRNPC over-expression by isotope tracing ^13^C manner. **(C)** The pH value of the culturing medium of GC cells with HNRNPC silencing or overexpression. **(D, E)** The extracellular acidification rate was tested after transfection of MKN74 cells with HNRNPC silencing or overexpression. **(F, G)** The oxygen consumption rate (OCR) was tested after transfection of MKN74 cells with HNRNPC silencing or overexpression. *p<0.05; **p<0.01. NS is "no significant".

To explore the function of HNRNPC on GC malignant phenotypes, the series of assays were performed. Firstly, the proliferation assay indicated that HNRNPC silencing repressed the proliferation and HNRNPC overexpression promoted it (**Figure 2A**). In our analysis, our data indicated that HNRNPC over/silencing was closely correlated to the lactate generation in the GC cells, thus the following assays was performed to investigate the lactate-related index. The lactate accumulation in the culture environment was detected and results indicated that HNRNPC silencing repressed the lactate quantity, and HNRNPC overexpression promoted the lactate (**Figure 2B**). Moreover, the HNRNPC silencing increased the pH value of the culturing medium, and HNRNPC overexpression reduced the pH value of the culturing medium (**Figure 2C**). To confirm the effect of HNRNPC, ECAR and oxygen consumption rate (OCR) were detected. ECAR of seahorse glycolytic stress tests demonstrated that the activation of glycolysis in GC cells was significantly reduced by HNRNPC silencing, in contrast to that of HNRNPC overexpression (**Figures 2D, E**). OCR of oxygen consumption rate results demonstrated that HNRNPC silencing enhanced mitochondrial respiration in GC cells, while HNRNPC overexpression reduced the mitochondrial respiration (**Figures 2F, G**). Conclusively, the results obtained from this study affirmed that HNRNPC positively fortified the aerobic glycolysis and lactate accumulation in GC.

### 3.3 Exogenous lactate accelerated the proliferation, oxaliplatin resistance and aerobic glycolysis in GC that inhibited by HNRNPC silencing

**Figure 3:**
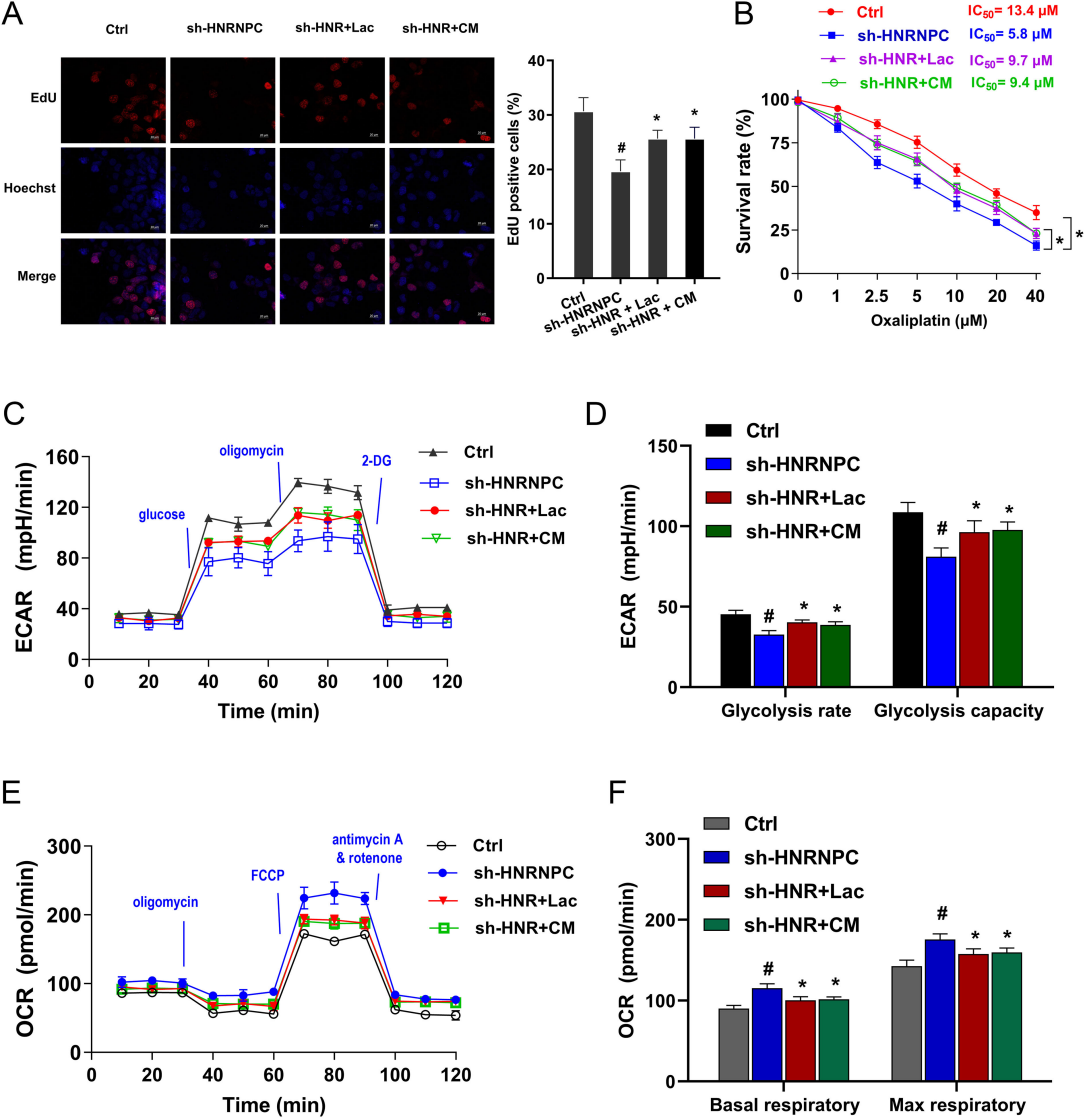
Exogenous lactate accelerated the proliferation, oxaliplatin resistance and aerobic glycolysis in GC that inhibited by HNRNPC silencing. **(A)** EdU assays were performed in the GC (MKN74) cells with stably HNRNPC silencing (sh-HNRNPC-1). Besides, the GC cells were also treated with exogenous lactate, including lactate (L-lactate) and conditioned medium (CM). **(B)** The oxaliplatin chemotherapy sensitivity test by CCK - 8 showed the half maximal inhibitory concentration (IC_50_) of GC cells toward oxaliplatin. **(C, D)** The ECAR analysis revealed the glycolytic capacity and glycolytic rate of GC cells with HNRNPC silencing and lactate and CM treatment. **(E, F)** The OCR analysis unveiled the basal and maximal respiratory rate levels of GC cells with HNRNPC silencing and lactate and CM treatment. ^#^p<0.05 relative to Ctrl group; *p<0.05, **p<0.01 relative to sh-HNRNPC group.

Given that previous findings showed the role of HNRNPC silencing on lactate inhibition, the further assays were performed to verify the interaction within HNRNPC and lactate in GC. The exogenous lactate was administrated to the culture to stimulate the GC cells, including lactate (L- lactate) and conditioned medium (CM). Carcinoma-associated fibroblasts (CAFs) is considered as one of the most abundant components in tumor and the major lactate source in the extracellular environment (19). CAFs-CM with elevated lactate level was prepared for culture of GC cells. The following assays monitored the GC cells’ response to HNRNPC/lactate treatment. Proliferation assay indicated that lactate and CM both recovered the inhibition by HNRNPC silencing (**Figure 3A**). The oxaliplatin chemotherapy sensitivity test showed that lactate and CM both promoted the half maximal inhibitory concentration (IC_50_) of GC cells toward oxaliplatin (**Figure 3B**). Thus, exogenous lactate could accelerate the oxaliplatin resistance of GC cells. ECAR analysis revealed that lactate and CM both promoted the glycolytic capacity and glycolytic rate of GC cells with HNRNPC silencing (**Figures 3C, D**). Then, the OCR analysis unveiled that lactate and CM both reduced the OCR at both basal and maximal respiratory rate levels of GC cells with HNRNPC silencing (**Figures 3E, F**). Conclusively, the results obtained from this study affirmed that exogenous lactate accelerated the proliferation, oxaliplatin resistance and aerobic glycolysis in GC that inhibited by HNRNPC silencing.

### 3.4 Lactate accelerated the ferroptosis resistance in GC with HNRNPC silencing

**Figure 4:**
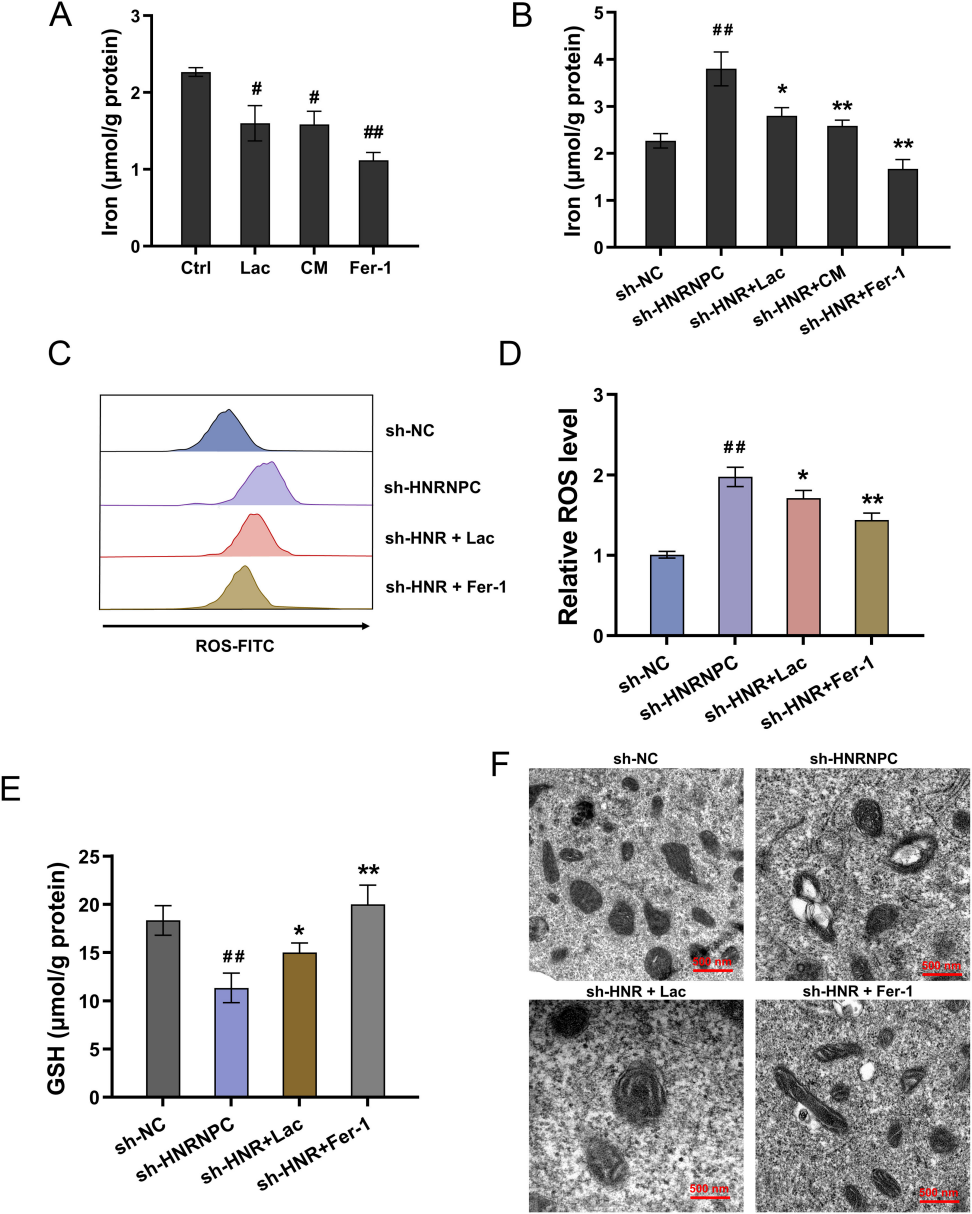
Lactate accelerated the ferroptosis resistance in GC with HNRNPC silencing. **(A, B)** The iron concentration was tested in GC cells (MKN74) with lactate (Lac), conditioned medium (CM), Ferrostatin-1 (Fer-1, 10 μM) and HNRNPC silencing (sh-HNRNPC). **(C, D)** The lipid ROS level and **(E)** GSH and **(F)** mitochondria morphology were tested in the GC cells with HNRNPC silencing (sh-HNRNPC), lactate (Lac) and Ferrostatin-1 (Fer-1, 10 μM). ^#^p<0.05, ^##^p<0.01 relative to Ctrl/sh-NC group; *p<0.05, **p<0.01 relative to sh-HNRNPC group.

Emerging literature are revealing the important role of iron-dependent cell death, also known as ferroptosis. Here, this study tried to investigate the role of HNRNPC/Lactate on iron and its relative ferroptosis. Iron deposition is related to intracellular accumulation of iron ions and mitochondrial metabolism. The lactate and CM administration could reduce the iron concentration accumulation, which was also consistent with the ferroptosis specific inhibitor Ferrostatin-1 (Fer-1) (**Figure 4A**). Moreover, HNRNPC silencing up-regulated the iron concentration accumulation, and the lactate, CM and Fer-1 co-administration reduced the iron concentration (**Figure 4B**). Moreover, HNRNPC silencing aggravated the ROS level (**Figures 4C, D**), reduced the GSH (**Figure 4E**) and exacerbated the mitochondrial injury (**Figure 4F**). Conclusively, the results affirmed that lactate accelerated the ferroptosis resistance in GC with HNRNPC silencing.

### 3.5 MCT1 was identified as the downstream target of HNRNPC

**Figure 5:**
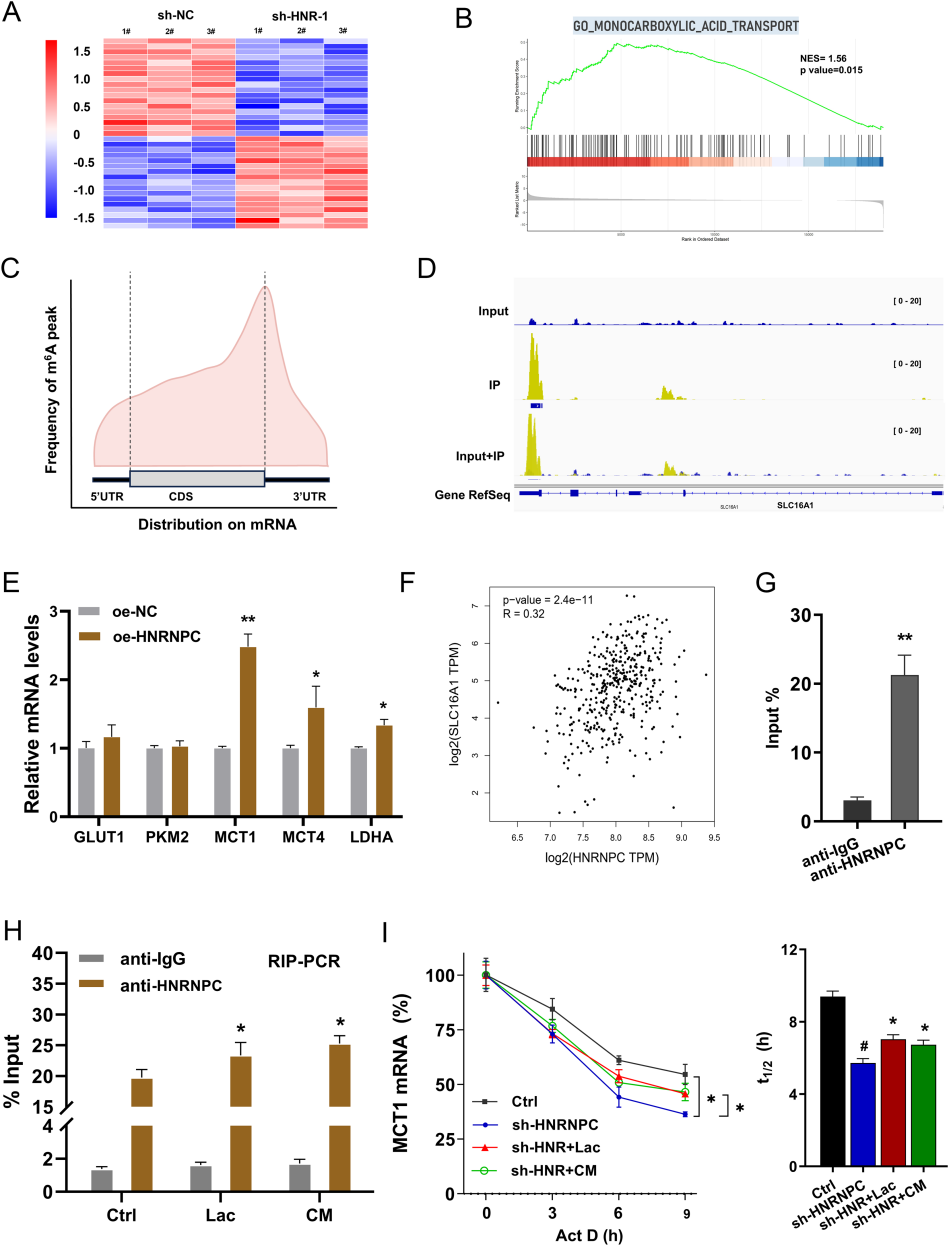
MCT1 was identified as the downstream target of HNRNPC. **(A)** Heatmap showed the RNA-Seq with sh-NC and sh-HNR-1#. **(B)** GSEA of the RNA-Seq. **(C)** The distribution of m^6^A peaks in GC transcript detected by MeRIP-seq. **(D)** The seq-data of the 3’-UTR of MCT1 gene was shown by the visualization tool (IGV, Integrative Genomics Viewer). **(E)** The several aerobic glycolysis related elements (GLUT1, PKM2, MCT1, MCT4, LDHA) were detected by RT-PCR. **(F)** In the public database (GEPIA, http://gepia.cancer-pku.cn/index.html), the correlation analysis within HNRNPC expression and MCT1 (SLC16A1 gene) level in STAD (Stomach adenocarcinoma) samples. **(G)** The RIP-PCR assay was performed to identify the interaction within the HNRNPC and MCT1 mRNA in GC cells. **(H)** The RIP-PCR assay was performed to explore the binding within HNRNPC and MCT1. **(I)** The RNA decay assay illustrated the MCT1 mRNA stability (half life time, t_1/2_) in GC cells with HNRNPC silencing, and the lactate or CM treatment. *p<0.05, **p<0.01 relative to NC or IgG group. ^#^p<0.05 relative to Ctrl group.

The previous results indicated that HNRNPC regulated the lactate accumulation in GC cells, thus the following assays were performed to explore the mechanism. The RNA-Seq in the sh-NC and sh-HNR-1# group was tested (**Figure 5A**). Gene set enrichment analysis (GSEA) of the RNA-Seq revealed that monocarboxylic acid transport was altered (**Figure 5B**). In the m^6^A sequencing data, the m^6^A modified profile was detected (**Figure 5C**). In the seq-data, the visualization tools showed that there was vital m^6^A modified site on the 3’-UTR of MCT1 gene (**Figure 5D**). Given the vital function of HNRNPC on aerobic glycolysis and lactate accumulation in GC, the following assays were performed to verify which element participated in the progression. Results indicated that MCT1 exerted the more remarkable alteration upon HNRNPC overexpression (**Figure 5E**). In the public database, the correlation analysis revealed that HNRNPC expression was positively correlated to the MCT1 (SLC16A1 gene) level in STAD (Stomach adenocarcinoma) samples (**Figure 5F**). RIP-PCR assay was performed and data revealed that HNRNPC significantly interacted with the MCT1 mRNA in GC cells (**Figure 5G**). Then, the RIP-PCR assay also illustrated that lactate and CM both increased the enrichment of MCT1 mRNA in incorporation within MCT1 mRNA and anti-HNRNPC (**Figure 5H**). The immunoprecipitation data revealed that exogenous lactate administration could enhance the binding within HNRNPC. For the mRNA fata of MCT1, the RNA decay assay illustrated that HNRNPC silencing reduced the MCT1 mRNA stability (half life time, t_1/2_), and the lactate and CM both increased the MCT1 mRNA stability (**Figure 5I**). Conclusively, the results obtained from this study affirmed that MCT1 was identified as the downstream target of HNRNPC, which was regulated by the lactate microenvironment.

### 3.6 HNRNPC targeted MCT1 to fortify the aerobic glycolysis and lactate accumulation in GC, thereby accelerating the ferroptosis resistance

**Figure 6:**
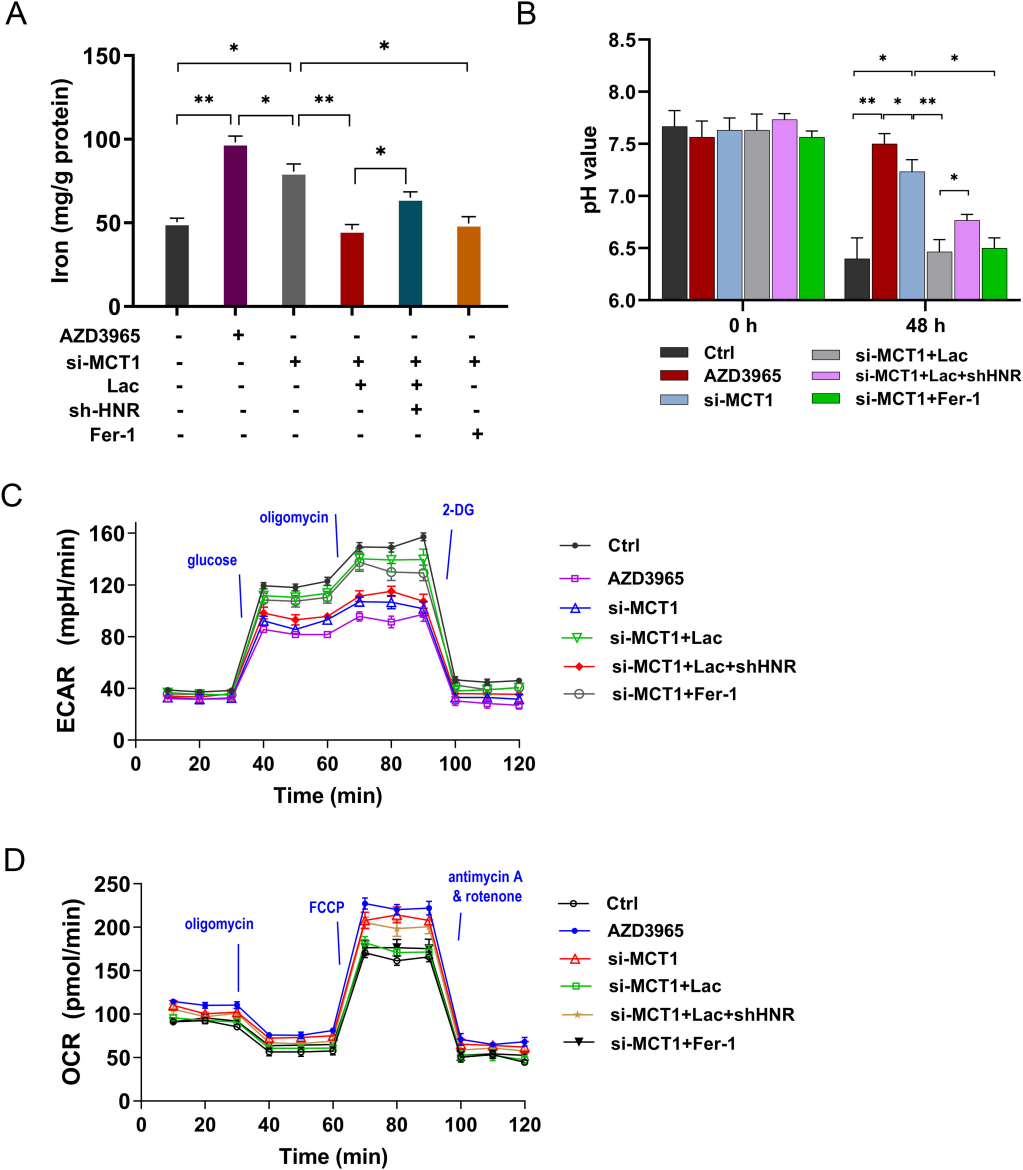
HNRNPC targeted MCT1 to fortify the lactate accumulation in GC, thereby accelerating the aerobic glycolysis and ferroptosis resistance. **(A)** The iron concentration was tested in GC cells (MKN74) with AZD3965 (MCT1 special inhibitor), MCT1 small interfering RNA (si-MCT1), L-lactate (Lac, 20 mmol/L), conditioned medium (CM), Ferrostatin-1 (Fer-1, 10 μM) and HNRNPC silencing (sh-HNRNPC). **(B)** The pH value of each group was tested. **(C)** The ECAR analysis revealed the glycolysis of GC cells. **(D)** The OCR analysis unveiled the respiratory rate levels of GC cells. *p<0.05, **p<0.01 relative to linked group.

Our previous findings showed that HNRNPC regulated the aerobic glycolysis and lactate accumulation and ferroptosis in GC. Besides, MCT1 was identified as the downstream target of HNRNPC. Thus, to verify the function of HNRNPC/MCT1 on the GC malignant phenotype, the rescue assays were performed in this part. The iron concentration, pH value, ECAR and OCR were respectively detected. MCT1 special inhibitor AZD3965 and MCT1 small interfering RNA (siRNA) up-regulated the iron concentration, increased the pH value and reduced the glycolysis (**Figures 6A–D**). Exogenous lactate and ferroptosis specific inhibitor Ferrostatin-1 (Fer-1) reduced these indexes that up-regulated by si-MCT1. These assays confirmed the role of HNRNPC, MCT1 and lactate on the GC aerobic glycolysis and ferroptosis resistance. Therefore, HNRNPC targeted MCT1 to fortify the aerobic glycolysis and lactate accumulation in GC, thereby accelerating the ferroptosis resistance.

### 3.7 HNRNPC silencing repressed the tumor growth of GC via MCT1

**Figure 7:**
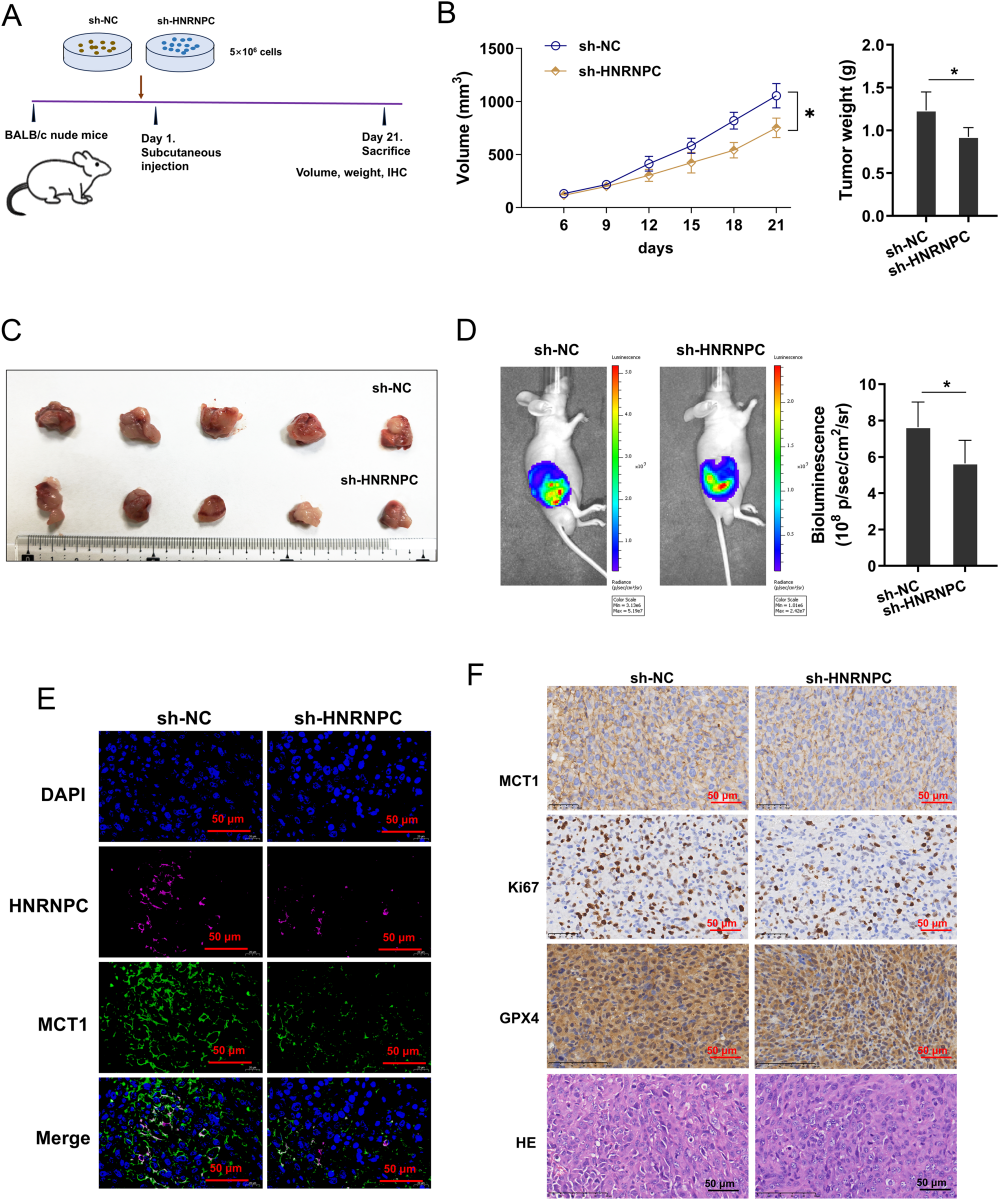
HNRNPC silencing repressed the tumor growth of GC via MCT1. **(A)** The xenograft mice assay was performed using BALB/c nude mice inoculated with MKN74 cells (sh-NC, sh-HNRNPC). **(B, C)** The tumor volume and weight were calculated in the HNRNPC silenced GC cells inoculation and control. **(D)** The growth was monitored and quantified by bioluminescence *In Vivo* Imaging System (IVIS). **(E)** The mIHC images of HNRNPC (pink), MCT1 (green) and DAPI (blue) in tumor tissue. **(F)** The immumohistochemical staining (IHC) of MCT1 protein and hematoxylin and eosin (HE) or Ki67, GPX4 staining in tissue. *p<0.05 relative to sh-NC group.

To explore whether HNRNPC influenced the tumor growth of GC *in vivo*, the xenograft mice assay was performed using nude mice (**Figure 7A**). In the tissue, the tumor volume and weight were reduced in the HNRNPC silenced GC cells inoculation (**Figures 7B, C**). Metastatic progression by noninvasive bioluminescence *In Vivo* Imaging System revealed that HNRNPC silencing reduced the tumor growth (**Figure 7D**). Besides, mIHC revealed that the MCT1 protein was also reduced in the HNRNPC silenced group (**Figure 7E**). Besides, the MCT1 level was also decreased in HNRNPC silenced GC cells inoculation (**Figure 7F**). Overall, these data indicated that HNRNPC silencing repressed the tumor growth of GC.

## 4 Discussion

Recent research has shown that lactate, a metabolite of glycolysis, plays a key role in the tumor microenvironment. lactate contributes to the invasion and metastasis of tumor cells by lowering the local pH, resulting in an acidic microenvironment (20). In addition, lactate could inhibit the function of immune cells, weaken the anti-tumor immune response, promote the survival and proliferation of tumor cells, and thus accelerate tumor progression.

In this study, the role of HNRNPC on the aerobic glycolysis and lactate accumulation was investigated in GC. Results indicated that HNRNPC positively fortified the aerobic glycolysis and lactate accumulation, which was verified in GC. Besides, HNRNPC silencing inhibited the proliferation, oxaliplatin resistance and aerobic glycolysis in GC cells. Moreover, the exogenous lactate (L-lactate, CM) accelerated these indexes that inhibited by HNRNPC silencing. Therefore, HNRNPC could regulate the lactate expression in the GC, and HNRNPC might promote GC progression through targeting lactate.

Ferroptosis is a novel cell death mechanism discovered in recent years, which is mainly caused by the accumulation of iron ions in cells. Unlike traditional forms of cell death such as apoptosis, necrosis, and autophagy, ferroptosis occurs in the binding of iron ions to mitochondrial proteins, interfering with cell metabolism, especially key enzymes in the tricarboxylic acid cycle (21). The concentration of iron ions triggers oxidative stress and protein aggregation, which leads to cell death. In GC, high-expressed METTL5 represses the Fe^2+^ accumulation to promote the GC immune evasion, which help GC cells escaping from CD8^+^ T cells’ killing effect. Besides, ferroptosis inhibitor Fer-1 reduces the antitumor immunity of CD8^+^ T cells (22). Therefore, the role of ferroptosis in tumorigenesis warrants investigation.

MCT1 (encoded by SLC16A1) is currently the focus of lactate metabolism research (23). The transport of MCT1 is pH-dependent, which can co-transport H^+^ and acidic intermediates of glucose metabolism (such as lactate, pyruvate, etc.). MCT1 is widely expressed in various tissues and organs, which could passively transport lactate according to the local lactate concentration gradient by flowing into or out of cells (24). Whether lactate could promote tumorigenesis by regulating ferroptosis is a question worth exploring. Here, this study revealed that MCT1 was identified as the downstream target of HNRNPC. Mechanistically, upregulation of HNRNPC promotes MCT1 mRNA stability, thereby activating the lactate efflux. HNRNPC targeted MCT1 to fortify the lactate accumulation, thereby accelerating the ferroptosis resistance in GC.

**Figure 8:**
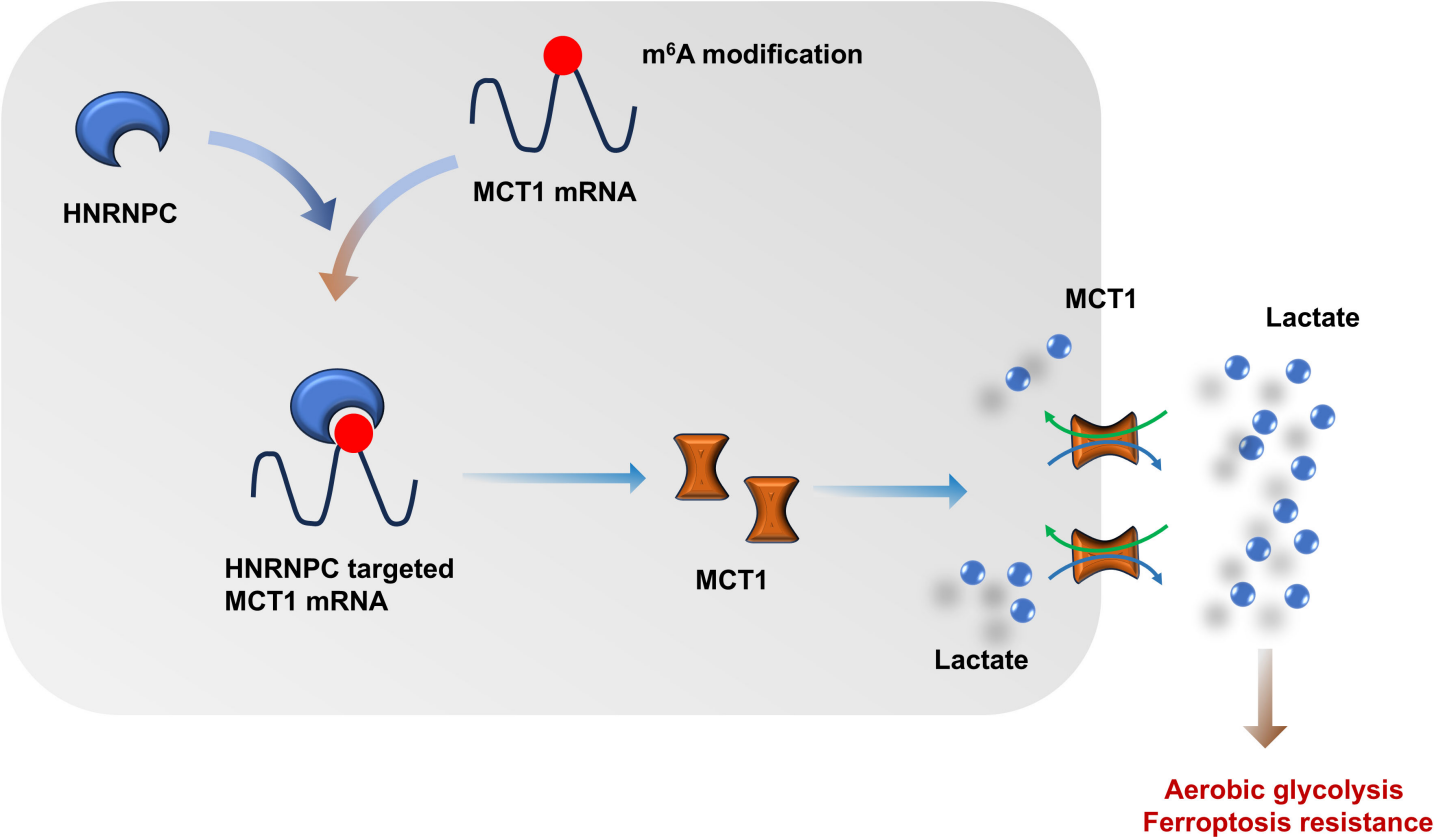
N^6^-methyladenosine (m^6^A) regulator HNRNPC fortifies the MCT1-mediated lactate accumulation in GC, thereby accelerating the aerobic glycolysis and ferroptosis resistance.

Overall, these findings revealed the critical role of HNRNPC on GC lactate accumulation and lactate-induced ferroptosis resistance in GC tumor microenvironment (**Figure 8**). The data revealed the importance of HNRNPC for lactate metabolism in GC tumor microenvironment, as well as the synergistic effect of HNRNPC on lactate-induced ferroptosis.

## Data Availability

The original contributions presented in the study are included in the article/**Supplementary Material**. Further inquiries can be directed to the corresponding author.
