# Supplementary figures and images for: Novel RNA-methylase HNRNPC promotes gastric cancer tumorigenesis by triggering the lactate-induced ferroptosis resistance
Source: Front Immunol. 2025 Sep 4;16:1612935. doi: 10.3389/fimmu.2025.1612935 (PMC12443684; doi:10.3389/fimmu.2025.1612935)

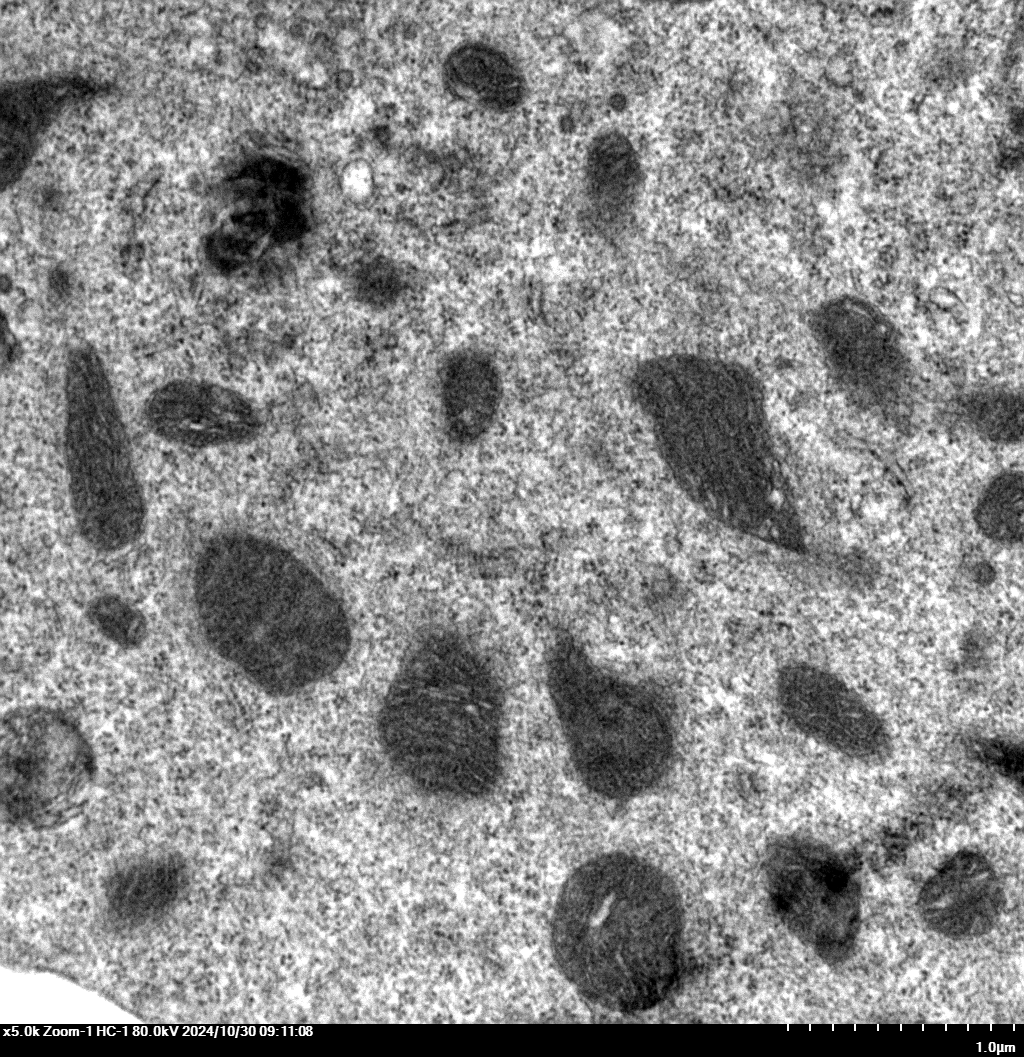

Supplement: Supplementary file 1 [file DataSheet1.zip › Raw data/Figure 4F TEM -1 .tif]

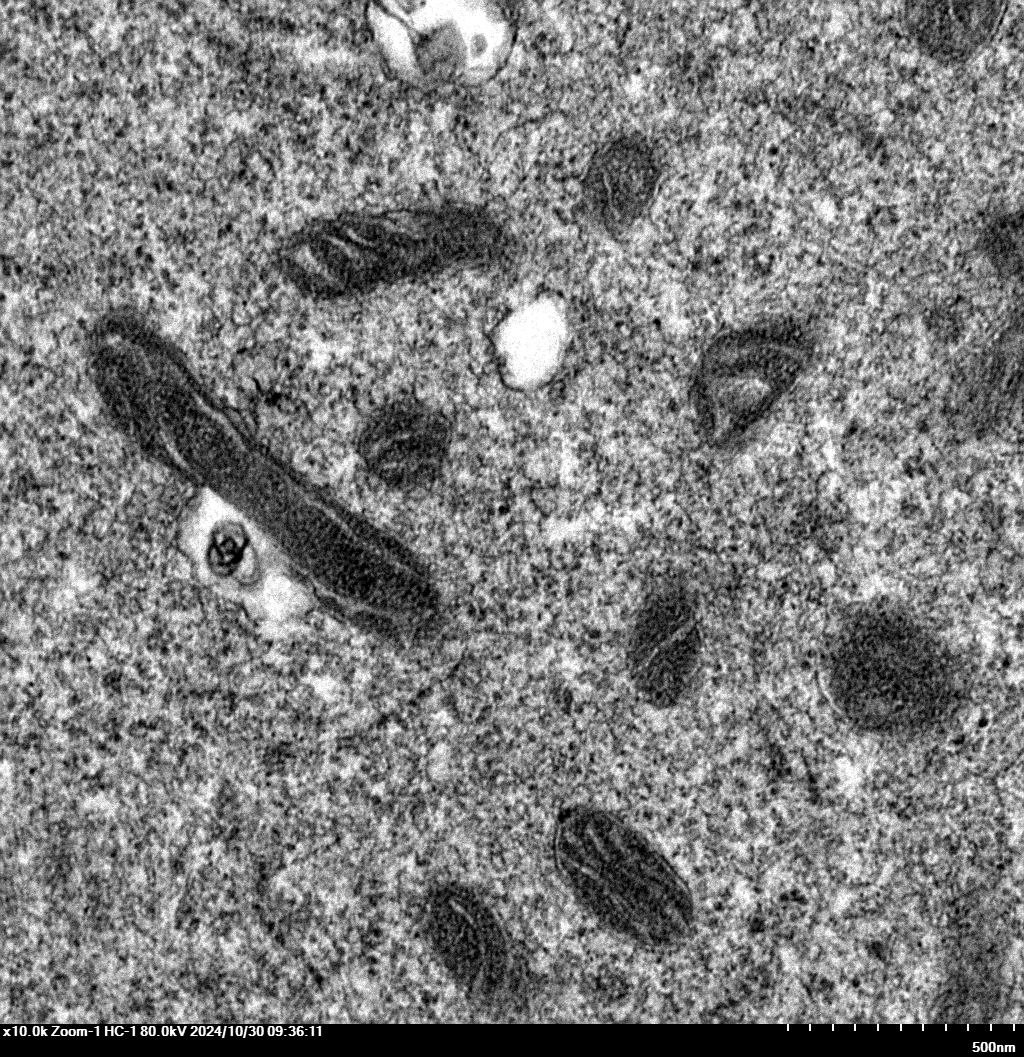

Supplement: Supplementary file 1 [file DataSheet1.zip › Raw data/Figure 4F TEM -2.tif]

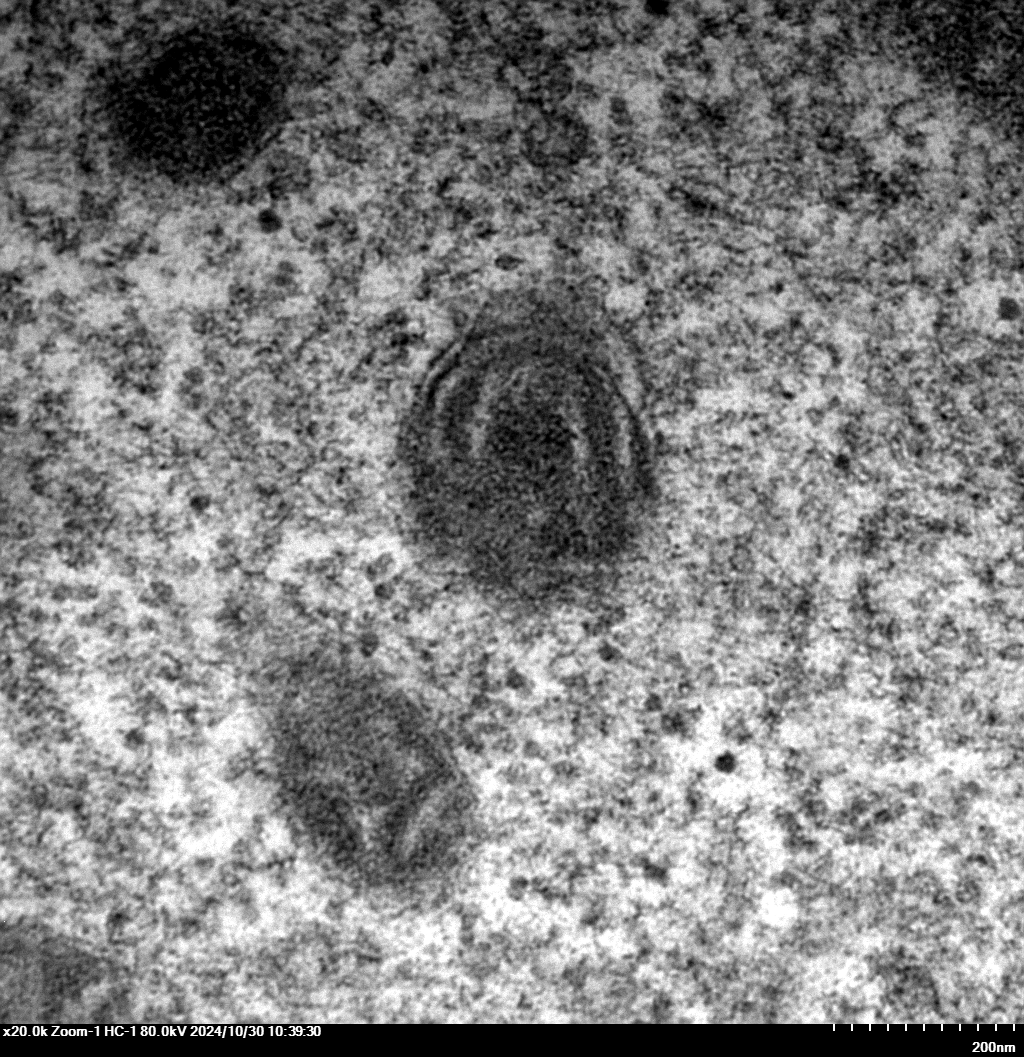

Supplement: Supplementary file 1 [file DataSheet1.zip › Raw data/Figure 4F TEM -3.tif]

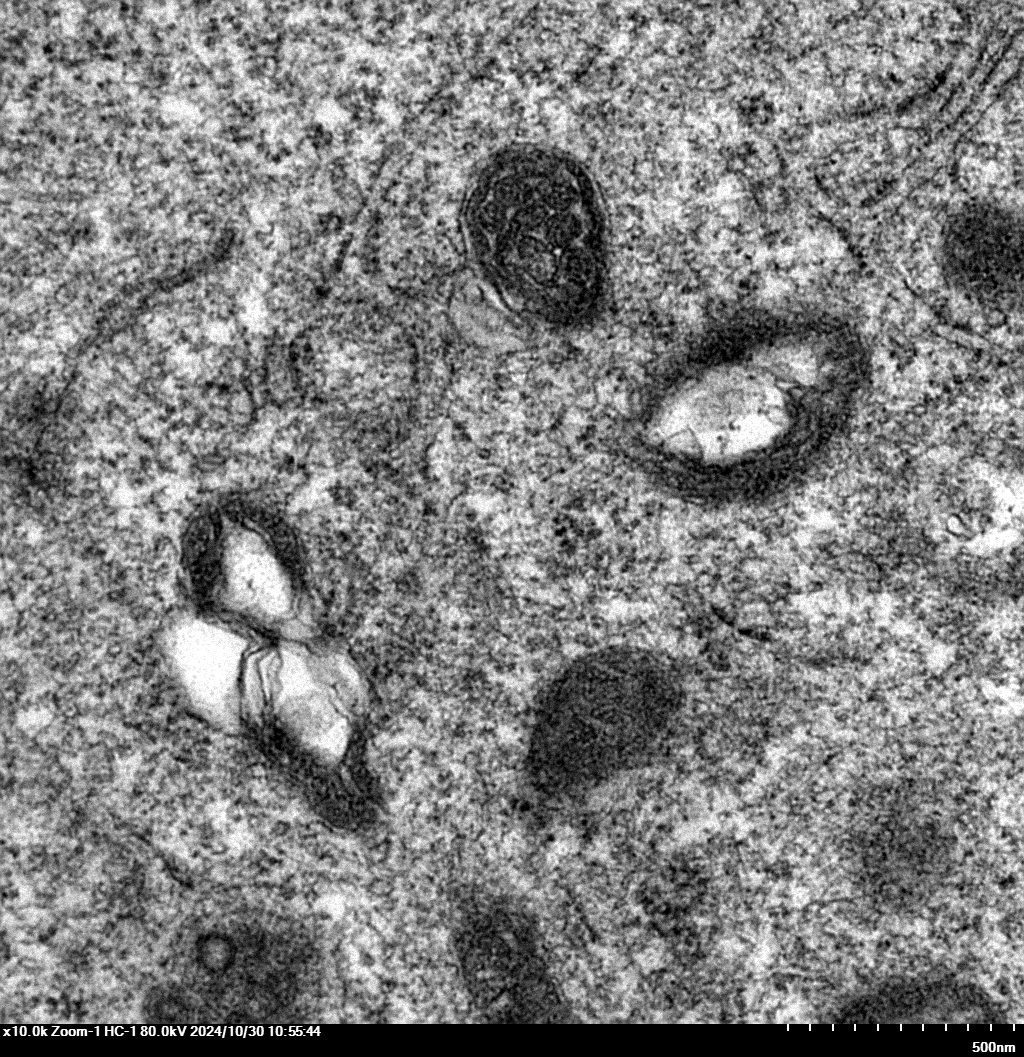

Supplement: Supplementary file 1 [file DataSheet1.zip › Raw data/Figure 4F TEM -4.tif]

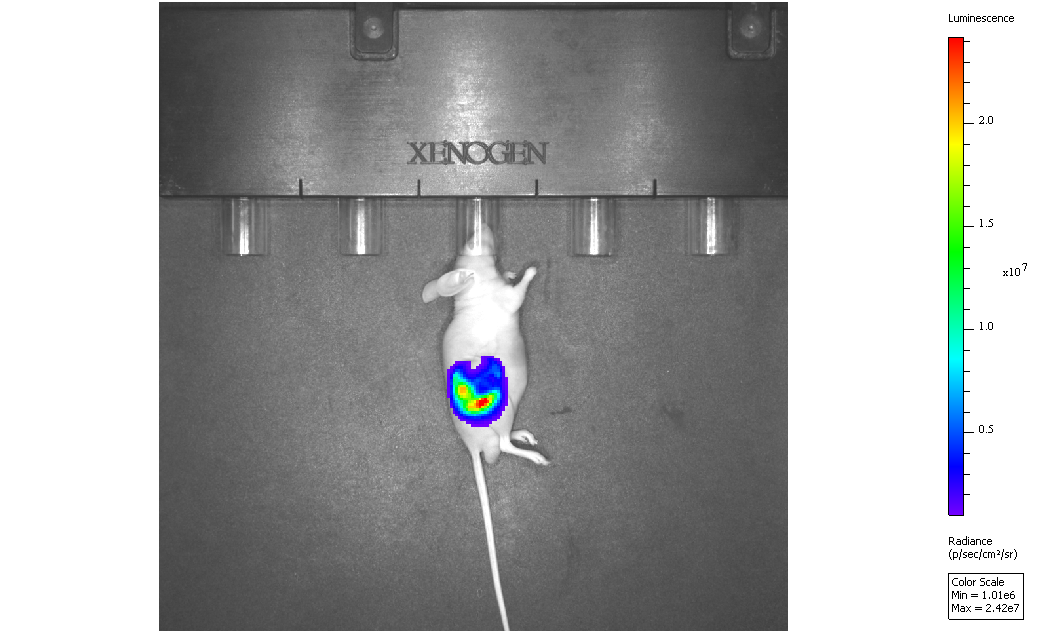

Supplement: Supplementary file 1 [file DataSheet1.zip › Raw data/Figure 7D Flu -1.png]

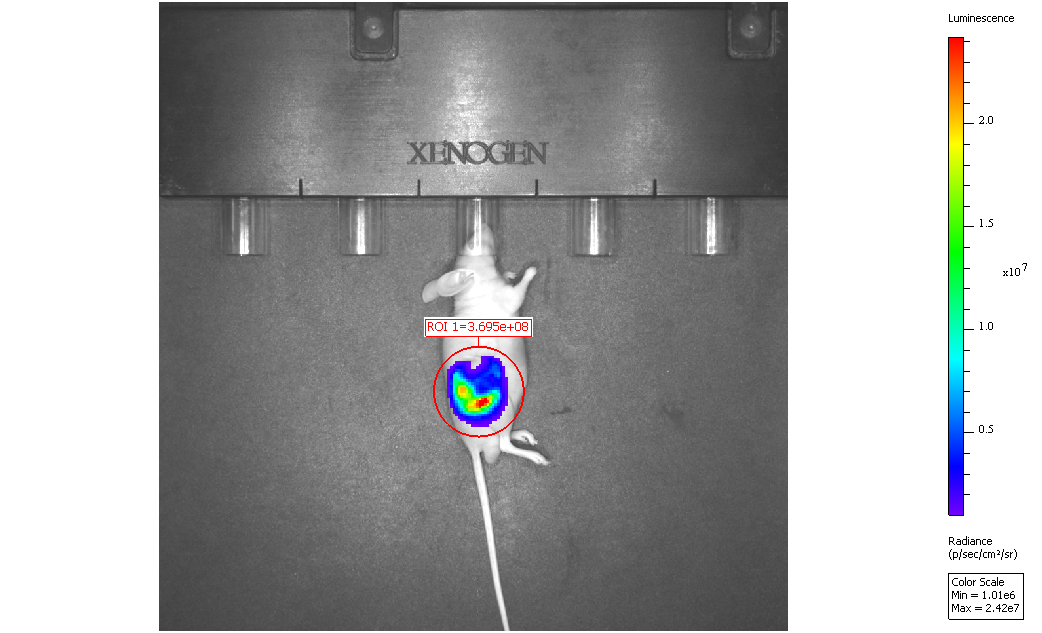

Supplement: Supplementary file 1 [file DataSheet1.zip › Raw data/Figure 7D Flu -2.png]

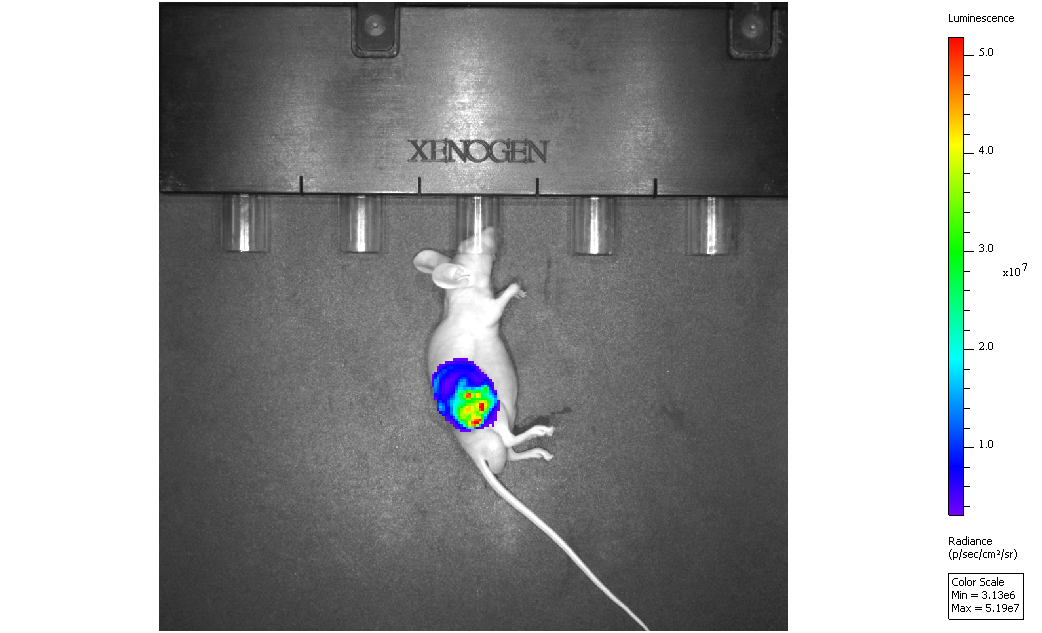

Supplement: Supplementary file 1 [file DataSheet1.zip › Raw data/Figure 7D Flu -3.png]

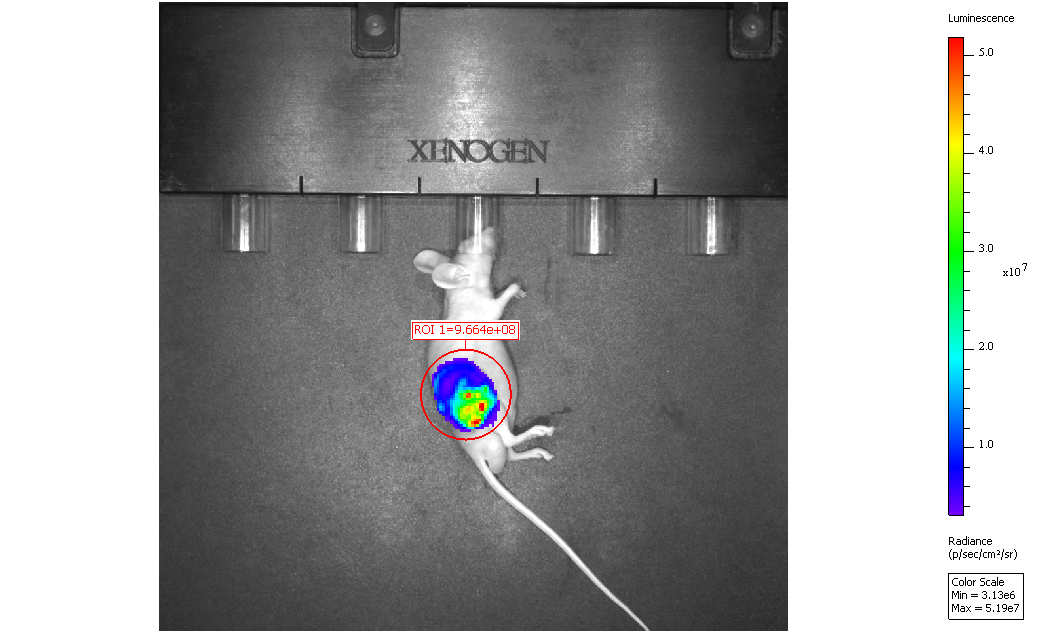

Supplement: Supplementary file 1 [file DataSheet1.zip › Raw data/Figure 7D Flu -4.png]

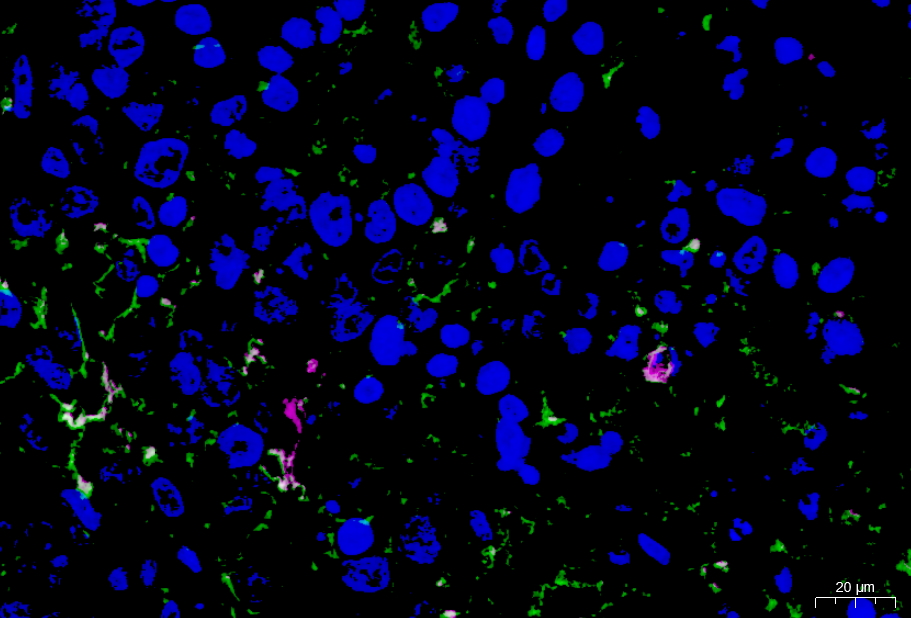

Supplement: Supplementary file 1 [file DataSheet1.zip › Raw data/Figure 7E mIHC -1 .jpg]

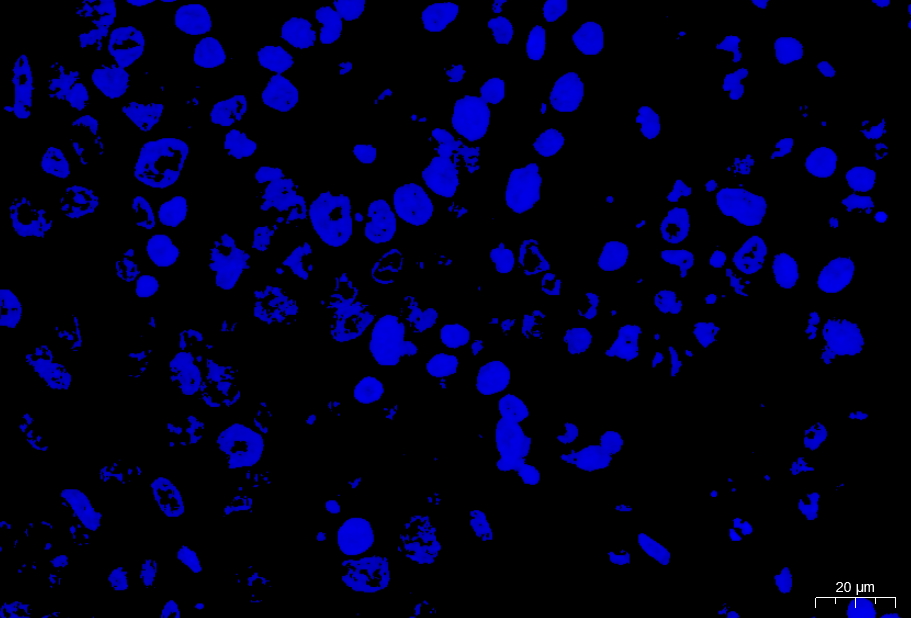

Supplement: Supplementary file 1 [file DataSheet1.zip › Raw data/Figure 7E mIHC -2 .jpg]

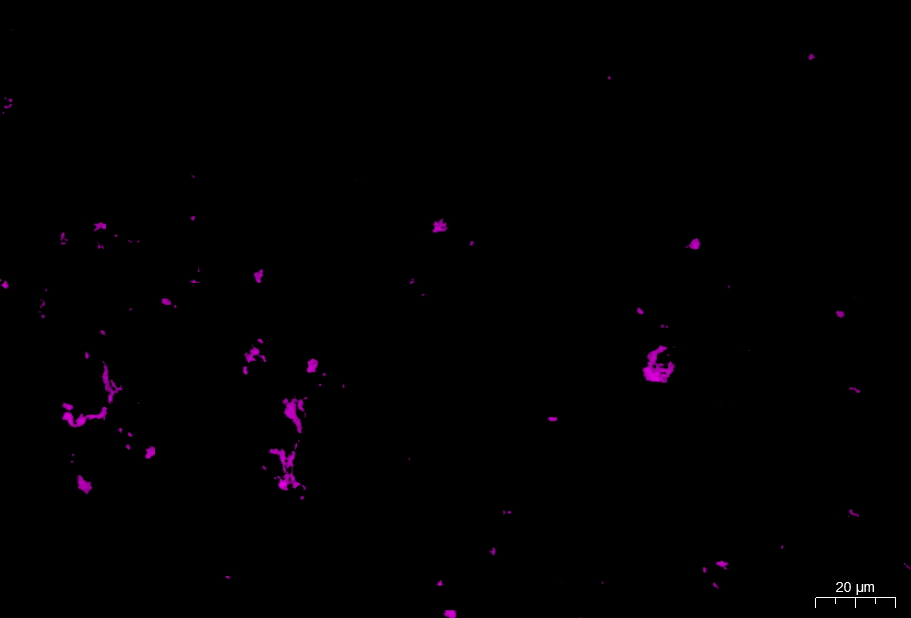

Supplement: Supplementary file 1 [file DataSheet1.zip › Raw data/Figure 7E mIHC -3.jpg]

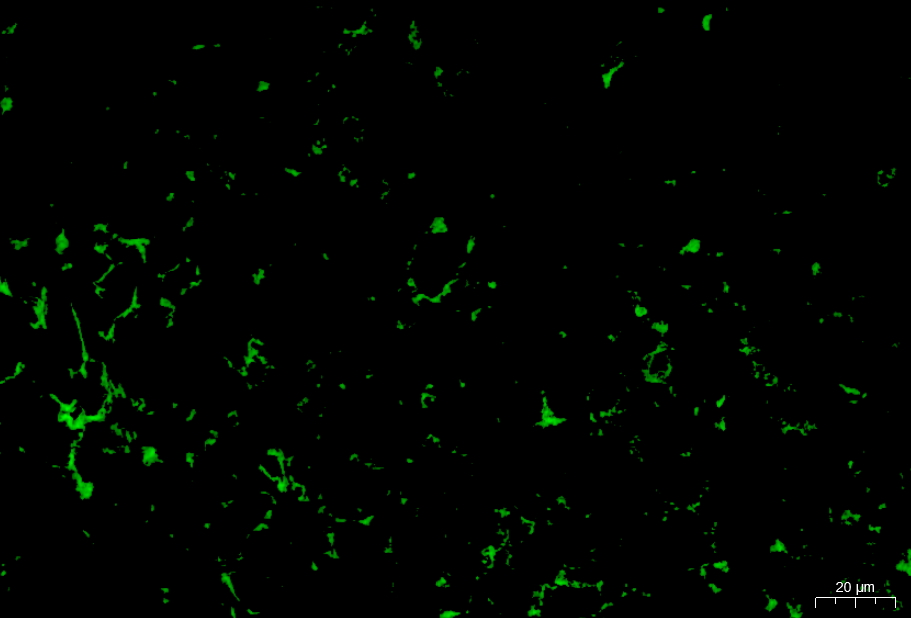

Supplement: Supplementary file 1 [file DataSheet1.zip › Raw data/Figure 7E mIHC -4 .jpg]

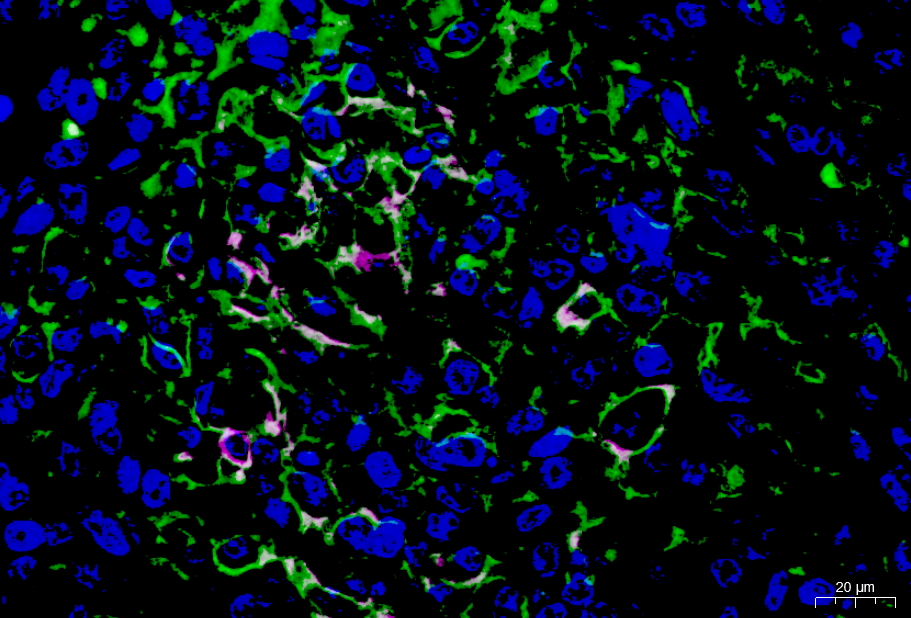

Supplement: Supplementary file 1 [file DataSheet1.zip › Raw data/Figure 7E mIHC -5 .jpg]

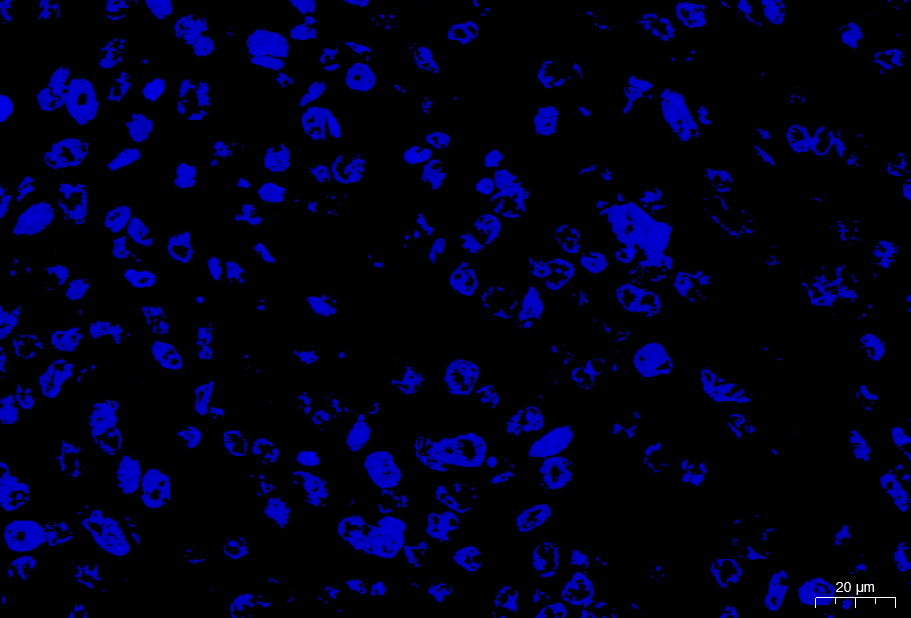

Supplement: Supplementary file 1 [file DataSheet1.zip › Raw data/Figure 7E mIHC -6 .jpg]

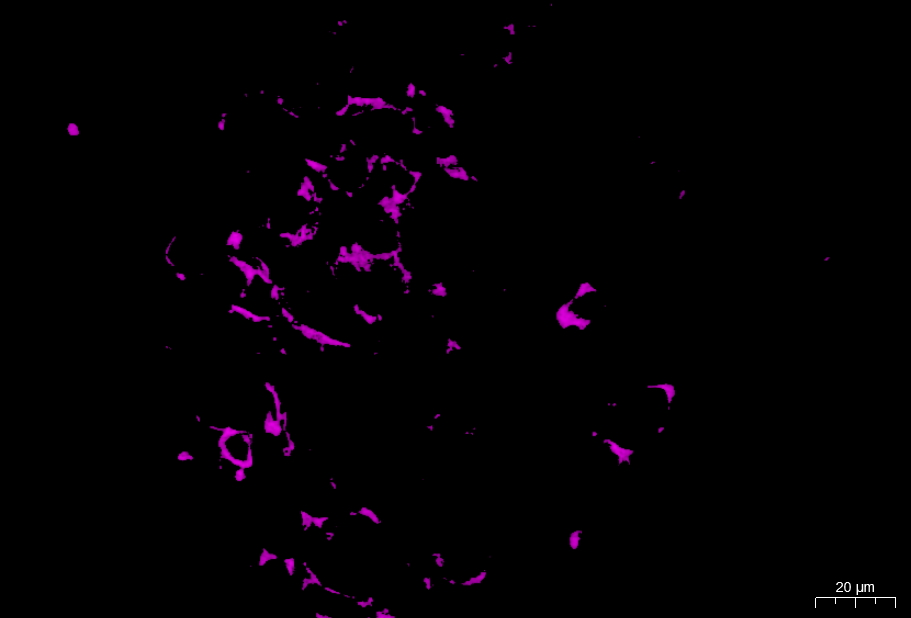

Supplement: Supplementary file 1 [file DataSheet1.zip › Raw data/Figure 7E mIHC -7 .jpg]

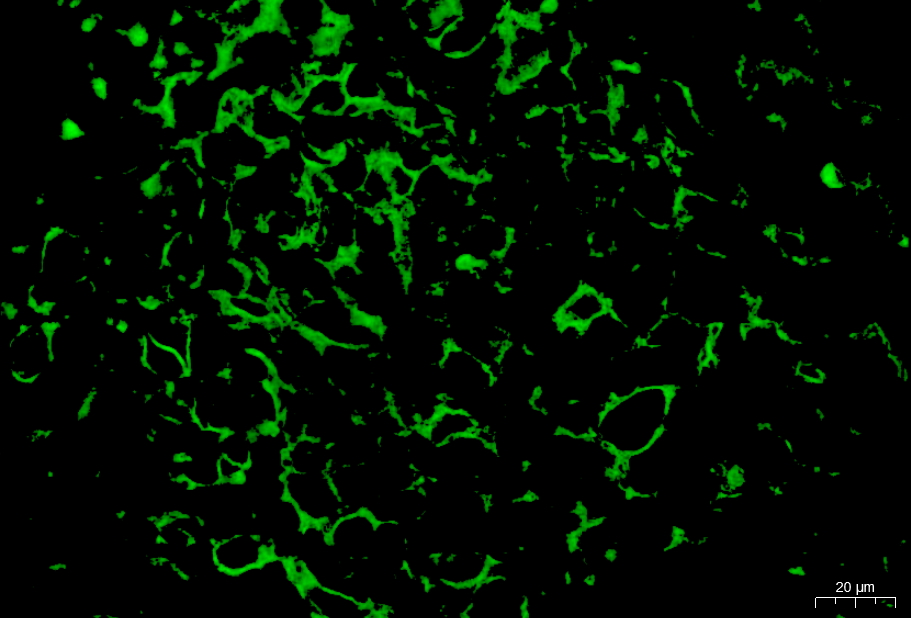

Supplement: Supplementary file 1 [file DataSheet1.zip › Raw data/Figure 7E mIHC -8 .jpg]
