# Supplementary material for: Novel RNA-methylase HNRNPC promotes gastric cancer tumorigenesis by triggering the lactate-induced ferroptosis resistance
Source: Front Immunol. 2025 Sep 4;16:1612935. doi: 10.3389/fimmu.2025.1612935 (PMC12443684; doi:10.3389/fimmu.2025.1612935)
Supplement: Supplementary file 2 [file Table1.docx]

**Table S1**. Primers and shRNAs.

| targets | sequences |
| --- | --- |
| HNRNPC | F, 5’-CCCTTCTCCGTCCCCTCTAC-3’  R, 5’-CCCGAGCAATAGGAGGAGGA-3’ |
| MCT1 | F, 5’- AGGTCCAGTTGGATACACCCC-3’  R, 5’- GCATAAGAGAAGCCGATGGAAAT-3’ |
| sh-HNRNPC-1 | 5’- GCGCTTGTCTAAGATCAAATT -3’ |
| sh-HNRNPC-2 | 5’- GCCTTCGTTCAGTATGTTAAT-3’ |
| GAPDH | F, 5’- GGAGCGAGATCCCTCCAAAAT-3’  R, 5’- GGCTGTTGTCATACTTCTCATGG-3’ |
| beta-actin | F, 5’- CATGTACGTTGCTATCCAGGC -3’  R, 5’- CTCCTTAATGTCACGCACGAT-3’ |
